# Comparison of fundus changes following silicone oil and sterilized air tamponade for macular-on retinal detachment patients

**DOI:** 10.1186/s12886-020-01523-9

**Published:** 2020-06-22

**Authors:** Yifan Zhou, Siqi Zhang, Hao Zhou, Min Gao, Haiyun Liu, Xiaodong Sun

**Affiliations:** 1 Department of Ophthalmology, Shanghai General Hospital (Shanghai First People’s Hospital), School of Medicine, Shanghai Jiao Tong University, Shanghai, China; 2 National Clinical Research Center for Eye Diseases, Shanghai, China; 3 Shanghai Engineering Center for Visual Science and Photomedicine, Shanghai, China; 4 Shanghai engineering center for precise diagnosis and treatment of eye diseases, Shanghai, China; 5 Shanghai Key Laboratory of Fundus Disease, Shanghai, China

**Keywords:** Retinal detachment, Pars plana vitrectomy, Silicone oil, Optical coherence tomography (OCT), Angiography

## Abstract

**Background:**

To investigate different tamponade effects of intravitreal silicone oil (SO) and sterilized air on macular vasculature and structure after successful retinal repair for macular-on rhegmatogenous retinal detachment (RRD) patients.

**Method:**

21 eyes (21 patients) with macular-on RRD underwent single pars plana vitrectomy following intravitreal SO or sterilized air (Gas) tamponade. Optical Coherence Tomography (OCT) and angiography were used to evaluate retinal layer thickness and flow density (FD) changes throughout the observation period of 12 weeks. Retinal layers were segmented into 7 sets: NFL, GCL + IPL, INL, OPL, ONL + IS, OS+RPE and BRM. Macular perfusion system was segmented into superficial and deep capillary plexus flow density (SCPFD, DCPFD), and choriocapillaries plexus flow density (CCPFD).

**Result:**

Compared to Gas tamponade, SO tamponade led to more decrease in both superficial and deep retinal blood flow during observation. NFL thickness was found to decrease in both Gas tamponade and SO tamponade eyes. SO tamponade resulted in more pronounced decrease which led to significant intergroup difference. Opposite changing trends were found in GCL + IPL and ONL + IS thicknesses due to different means of tamponade. SO tamponade caused thicknesses of these two segmented layers to decrease, which led to significant intergroup differences. SO tamponade also led to more decrease in INL, OPL thicknesses. No significant intergroup difference of choroidal thickness was observed.

**Conclusion:**

Compared to gas, silicone oil could have more negative tamponade effects on both fundus vasculature and structure.

## Background

Retinal detachment (RD), characterized by a separation of the sensory retina from the retinal pigment epithelium (RPE), is one of the leading causes for permanent vision loss. Among its subcategories, rhegmatogenous retinal detachment (RRD) is the most common one with annual incidence to be about 10.5 people per 100,000 population [1]. One of the most popular and effective surgical options is pars plana vitrectomy (PPV), which could achieve amazing reattachment rates in RRD patients. According to clinical observations, researchers have demonstrated that the maintenance of macular structure might be related to the postoperative visual outcome [2–5]. However, concerns have been raised that patients who seemed to have no macular abnormalities, confirmed by means of conventional Optical Coherence Tomography (OCT), could also suffer from progressive visual impairment after successful reattachment surgery. A series of reports have indicated the presence of unexplained severe visual damage, especially after intravitreal silicone oil (SO) use, which is also known as: Silicone Oil-Related Visual Loss (SORVL) [6–8].

For fundus surgeons, SO and gas are frequently-used intravitreal tamponade mediums for retinal repair. As a biochemically inert polymer, SO is widely used in vitreoretinal surgery and was considered to be well-tolerated and not threatening to retinal physiology [9–11]. However, some researchers have noticed potential harmful effects of silicone oil to retinal structure [12, 13]. Moreover, in RRD patients without macular involvement, incidence of unexplained vision loss after SO tamponade is by far higher than that after gas tamponade [5, 14]. Recent studies also considered that visual abnormalities like central scotoma, decreased foveal sensitivity and macular dysfunction might also be related to intravitreal SO application [6–8, 15]. Detailed fundus imaging and observation on SO tamponade effects on fundus structure could be essential to understand the underlying mechanisms.

To our knowledge, most related studies were designed as cross-sectional clinical observations. Negative tamponade effects of SO have been described such as thickness change in specific retinal layers [16, 17] and choroidal thinning [18]. However, few studies focused on the demonstration of different tamponade effects owing to different intravitreal tamponade materials, or included follow-up observations. On the other hand, in most previous researches, the study designs were widely divergent. Patients with macular-on or macular-off RRD were often mixed up for analysis. Different intraoperative pathological conditions, especially the preoperative macular status, may have a much larger contribution to differences on OCT and OCTA metrics than tamponade choices or surgical options. For fundus structure, various retinal cells underwent irreversible cell death after detachment [19]. For fundus vasculature, macular vasculature might maintain intact in macular-on RRD patients, rather than macular-off RRD patients [20]. Furthermore, various surgical options: combined operation with cataract surgery during retinal repair [21, 22], either or both scleral buckling and PPV for retinal reattachment [15, 21–26] and multifarious intravitreal tamponade choices [15, 17, 22, 23], could all have impact on fundus structure and vasculature. Thus, the comparison of different tamponade effects of silicone oil and other intravitreal tamponade deserve further study with rigorous design, especially in RRD patients without macular involvement.

To eliminate the influence of preoperative macular status and the impact of different surgical options on macular vasculature and structure, we prudently chose macular-on RRD patients, who only received single PPV for retinal reattachment in the present study. Detailed OCT/OCTA examinations on postoperative fundus vasculature and structure changes were performed during the follow-up observations. We focused on the intergroup comparison of fundus changes between SO tamponade and air tamponade eyes, and we intended to demonstrate whether SO tamponade could lead to more macular abnormalities.

## Methods

### Patients

**Fig. 1:**
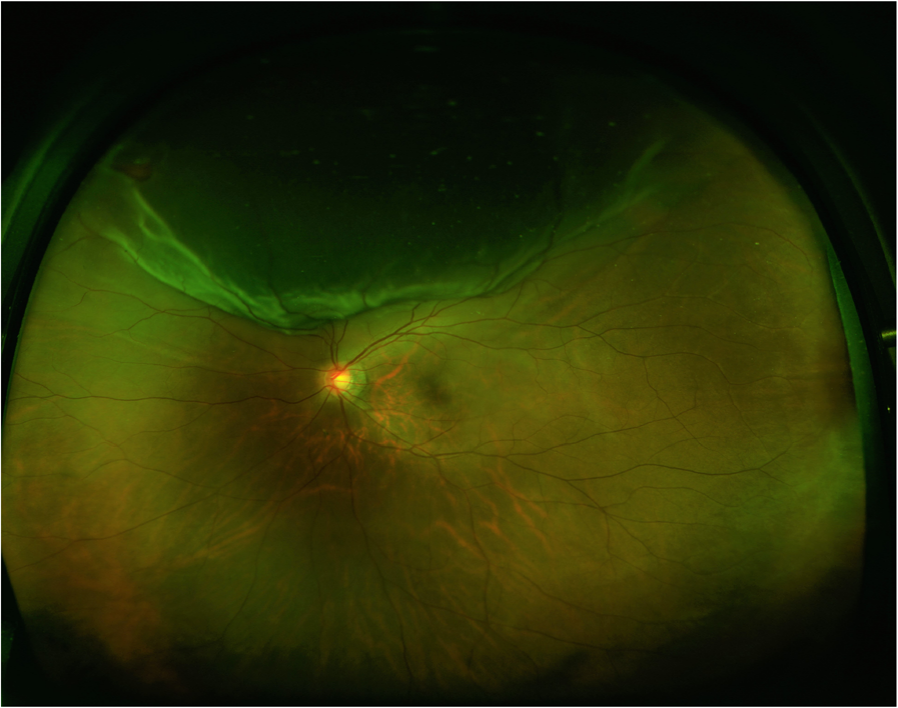
This macular-on RRD patient had a relatively intact macular structure, 1.5 quadrants of detachment, and a horseshoe shaped tear at superonasal quadrant showed on the fundus photograph

This retrospective, single-center study included 21 patients of the inpatients Department of Ophthalmology, Shanghai General Hospital Affiliated to Shanghai Jiao Tong University, from October 2017 to March 2019. This study adhered to the tenets of the Declaration of Helsinki and was approved by the Ethics Committee of Shanghai General Hospital affiliated to Shanghai Jiao Tong University. All patients suffered from primary RRD without macular involvement (Fig. 1). Exclusion criteria included macular involvement in detachment, proliferative vitreoretinopathy over B level, previous history of intraocular surgery, sign of epiretinal membrane, macular hole, macular degeneration or other macular disorders, high myopia (axial length>26 mm), secondary glaucoma, lens turbidity or massive vitreous hemorrhage, persistent subretinal fluid and intraretinal cystic space. Patients with diabetes mellitus, poorly controlled hypertension or other severe systemic vascular diseases were also excluded.

### Surgery

Standard three-port 23-gauge PPV was performed using the Alcon Constellation system (Alcon Laboratories, Inc., Fort Worth, TX, USA) under retrobulbar anesthesia by the same surgeon (Dr. Haiyun Liu). Preoperative macular status, extent of RRD (number of quadrants) and number of retinal tears were recorded before surgery, and double confirmed during the procedure. Before surgery, adequate doctor-patient communications were carried with each of our patients. The choice for type of tamponade was first discussed during the conversation, mainly based on intraocular pathological condition, and was finally decided by the surgeon during the operation. Intravitreal tamponade was used after drainage of sub-retinal fluid during air-fluid exchange. 7 patients with intravitreal SO tamponade were defined as Silicone Oil (SO) group, and 14 patients with intravitreal sterilized air tamponade were defined as Gas group. Usually, sterilized air could be spontaneously absorbed in 2 weeks. Upon the first postoperative follow-up visit (at 2 weeks), patients of the Gas group actually had no remaining intravitreal air tamponade. But for ease of expression, we denominated the ‘Gas/SO group’ and ‘Gas/SO tamponade’ in the present study, based on intravitreal tamponade choices during surgery. No patient underwent combined cataract surgery during the procedure. SO removal was performed around 4 months after retinal reattachment surgery. No patient suffered from recurrent retinal detachment.

### Retinal and choroidal vascular layer imaging

**Fig. 2:**
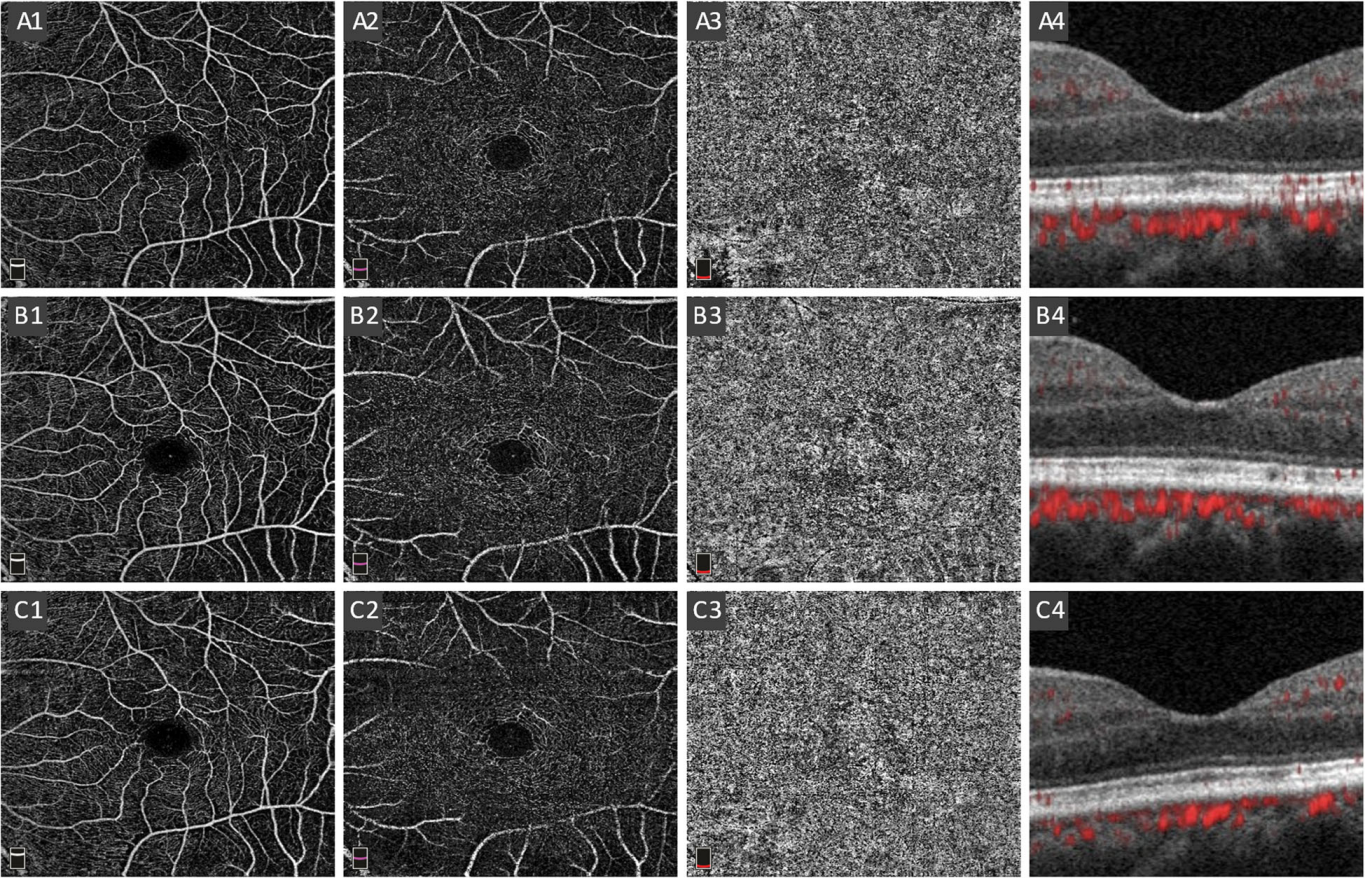
A1–3, B1–3 and C1–3 showed SCPFD, DCPFD and CCPFD in one RRD eye after successful retinal repair at three postoperative follow-up time points (2 weeks, 6 weeks and 12 weeks) respectively. A4, B4&C4 showed macular ultrastructure with blood flow signal

OCT-angiogram imaging was performed using the RTVue XR Avanti device (AngioVue software, version 2017.1.0.155; Optovue Inc., Fremont California, USA) with the Angio Retina mode (6 × 6 mm, blood flow density) by the same operator (H, Zhou). The technique including the SSADA method has been previously described [26, 27]. The software automatically segmented the vasculature into three layers: superficial capillary plexus (SCP), deep capillary plexus (DCP), and choriocapillaris plexus (CCP) (Fig. 2). The segmentation of the capillary plexus was checked before any data measurement was recorded. An experienced doctor (S, Zhang) and technician (H, Zhou) independently reviewed the images and occasionally corrected the segmentation lines when necessary. For each layer, flow density (FD) was calculated separately in three annulus regions: fovea (1 mm), parafovea (1–3 mm) and perifovea (3-6 mm) according to the ETDRS grids. The images with poor image quality were excluded based on one or more of the following criteria: low signal strength index (< 60), presence of blink artifacts, poor fixation leading to motion or doubling artifacts, and media opacities obscuring the view of the vasculature.

### Retinal and choroidal layer imaging

**Fig. 3:**
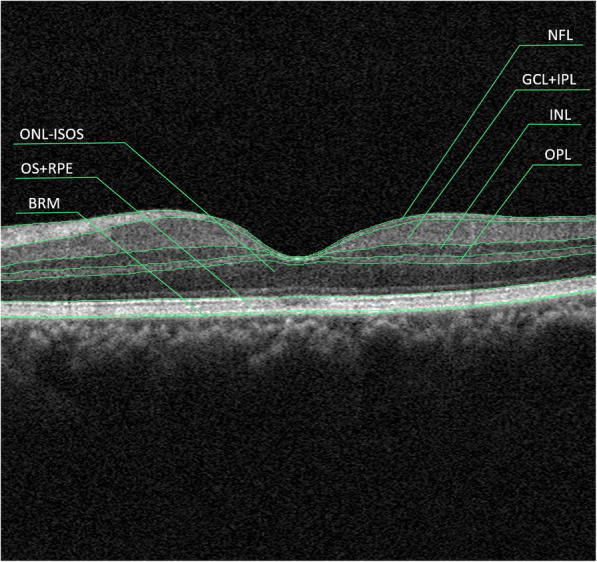
Sketch of automatic retinal layers segmentation produced by AngioVue software

With the automatic segmentation of retinal layers by RTVue XR Avanti device, we got seven sets of retinal layer thickness data for analyzation in the present study: NFL (nerve fiber layer), GCL + IPL (ganglion cell layer and inner plexiform layer), INL (inner nuclei layer), OPL (outer plexiform layer), ONL + IS (outer nuclei layer and inner segment), OS+RPE (outer segment and retinal pigment epithelium) and BRM (Brunch’s membrane) (Fig. 3). Choroidal thickness was obtained with the automatic built-in software of the SS-OCT device (Topcon FastMap, version 10.13.003.06, Topcon Medical Systems). All patients underwent three times of postoperative examinations at 2 weeks, 6 weeks and 12 weeks after surgery.

### Data analysis

All statistical analyses were performed using Python (version 3.7.3, Python Software Foundation). All values were given as mean ± SD. To demonstrate different tamponade effects, we emphasized on the changes in FD and layer thickness between two adjacent time points. Intergroup comparison of these changes between two tamponade groups was carried out using independent-samples t test. Values of *p* < 0.05 were considered to be statistically significant.

## Results

All 21 patients underwent three times of postoperative examinations. The mean age of our patients was 53.86 ± 8.25 years. The mean duration of presumed RD (duration of corresponding visual symptoms) before surgery was 11.19 ± 4.1 days. The mean axial length was 24.06 ± 0.68 mm. Demographics and clinical characteristics of different subgroups were shown in Table 1. No significant difference of mean age, gender, axial length or symptom duration before surgery was found between SO group and Gas group. The intraocular pathological conditions of retinal detachment had some differences between SO and Gas groups. Although the number of retinal tears had no significant difference between these two groups, the SO group had relatively larger extent of retinal detachment than the Gas group (*p* = 0.017).

**Table 1:**
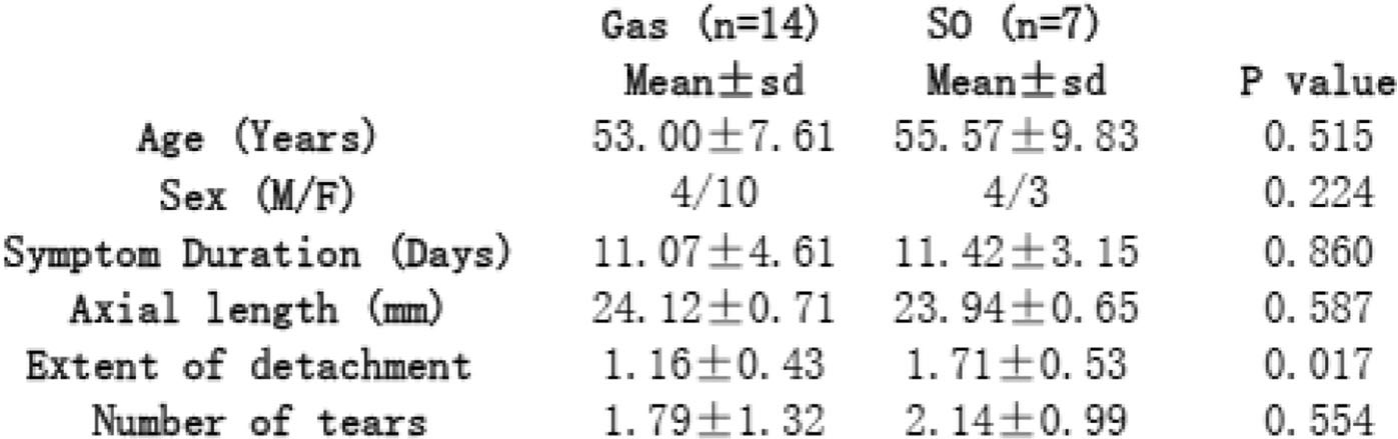
Demographic and clinical characteristics of the study population

To describe different tamponade effects, Tables 2 and 3 showed intergroup comparison of the changes of all evaluation indicators. To illustrate these changing values, Tables 4 and 5 presented all the measured values. During our observation, no significant result of intergroup comparison of choroidal thickness was found, thus, related data was not shown in Tables.

**Table 2:**
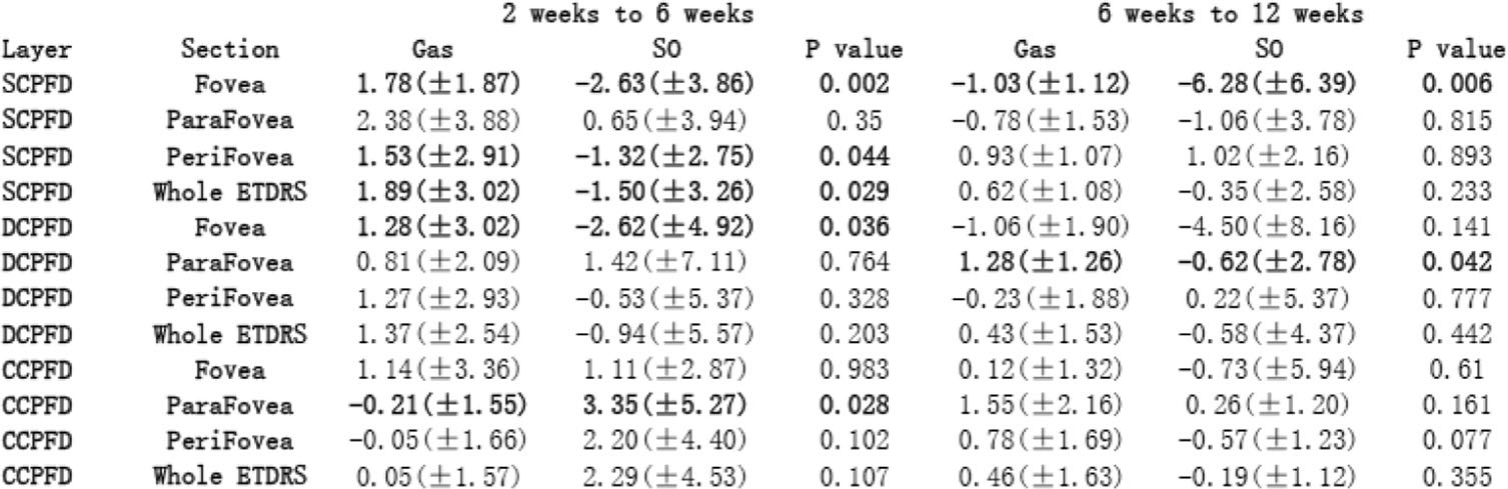
Intergroup comparison of fundus vasculature changes

**Table 3:**
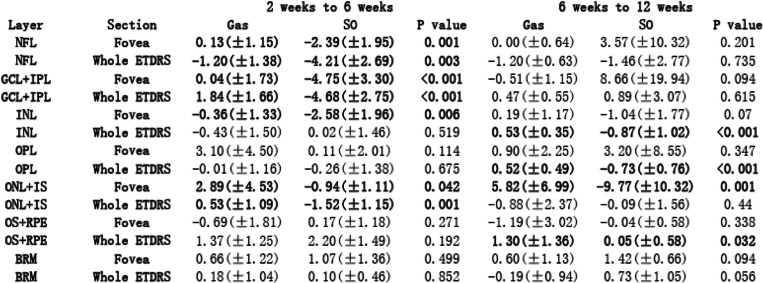
Intergroup comparison of fundus structural changes

**Table 4:**
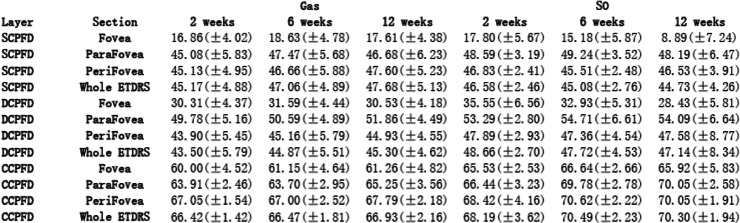
Measured OCTA data displayed to illustrate postoperative fundus vasculature change

**Table 5:**
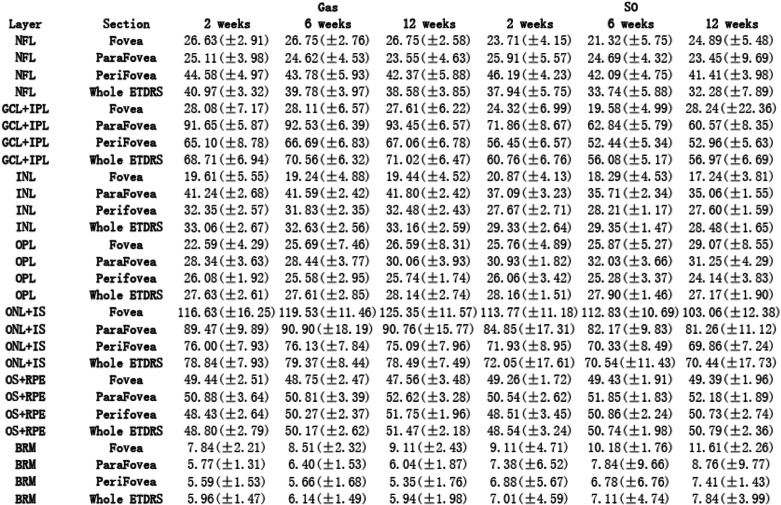
Measured OCT data displayed to illustrate postoperative retinal layer thickness changes

### Fundus vasculature

From 2 weeks to 6 weeks, we found opposite changing trends of SCPFD in Gas group and SO group, which led to significant intergroup differences (Table 2). SO tamponade led to more decrease in SCPFD, while the blood flow had a slight increase in Gas tamponade eyes. Similarly, foveal DCPFD also had opposite changing trends between Gas group and SO group (Gas: 1.28 ± 3.02, SO: − 2.62 ± 4.92, *p* = 0.036, Table 2). Furthermore, parafoveal CCPFD showed more increase in SO tamponade eyes which also led to significant intergroup difference (Gas: − 0.21 ± 1.55, SO: 3.35 ± 5.27, *p* = 0.028, Table 2).

From 6 weeks to 12 weeks, we found decreased foveal SCPFD in both SO and Gas tamponade eyes. SO tamponade eyes had more decrease in blood flow which led to significant intergroup difference (Gas: − 1.03 ± 1.12, SO: − 6.28 ± 6.39, *p* = 0.006, Table 2). Compared to gas tamponade, SO tamponade also resulted in more decrease in parafoveal DCPFD, which led to significant difference (Gas: 1.28 ± 1.26, SO: − 0.62 ± 2.78, *p* = 0.042, Table 2).

No significant result was found in other analysis of retinal or choroidal capillary plexus flow density changes during observation.

### Retinal layers and choroidal thickness

From 2 weeks to 6 weeks, NFL thickness was found to decrease in both Gas tamponade and SO tamponade eyes (Table 3). SO tamponade resulted in more pronounced decrease which led to significant intergroup differences. Opposite changing trends were found in GCL + IPL and ONL + IS thicknesses due to different means of tamponade. SO tamponade led to more decrease in these two segmented layers, which also led to significant intergroup differences. Foveal INL thickness had more decrease in SO tamponade eyes as well (*p* = 0.006, Table 3).

From 6 weeks to 12 weeks, SO tamponade led to more decrease in INL and OPL thicknesses, while gas tamponade led to a slight increase, which led to significant intergroup difference (Table 3). Foveal ONL + IS thickness also showed opposite changing trends due to different tamponades. SO tamponade led to more decrease in ONL + IS thickness, while Gas tamponade led to an increase (*p* = 0.001, Table 3). OS+RPE thickness had more increase in Gas tamponade eyes compared to SO tamponade eyes (*p* = 0.032, Table 3).

No significant result was found in other analysis of retinal or choroidal layer thickness changes during observation.

## Discussion

As described before, the present study had a rigorous design. Before us, few studies focused on the postoperative macular changes in macular-on RRD patients and different tamponade effects due to various intravitreal tamponade choices. We made prudent criteria to avoid potential influence caused by preoperative macular status and surgical options on macular area. Furthermore, we emphasized on the changing value of all OCT/OCTA evaluation indicators during follow-up. Thus, compared to previous studies, we believe that our work could be more reliable in demonstrating different tamponade effects of silicone oil and sterilized air on postoperative fundus change.

It is unethical to randomly choose intravitreal SO or sterilized air as intravitreal tamponade. The criterion of the endotamponade selection was based on surgeon’s comprehensive estimation of intraocular pathological conditions (tear size/tear number/tear location/detachment extent etc.). In the present study, we tried to pick up patients with similar intraocular pathological conditions. The SO group had relatively larger extent of detachment than the Gas group, while the number of retinal tears and symptom duration had no significant intergroup difference (Table 1). More importantly, the macular area was not involved in detachment. Macular vasculature could remain intact in eyes with macula-on RRD [20]. Thus, we believe that our results could represent the true different tamponade effects between the two intravitreal tamponade materials without being much influenced by other factors. The best time for SO removal is yet to reach a consensus in practice. The removal of SO is recommended to reduce potential SO-related complications such as cataract, glaucoma, and keratopathy. As routine practice for our ophthalmic clinical center, anatomically well-reattached retina based on OCT examination and no sign of progressive PVR after PPV for RRD patients over 3 months indicate SO removal. Thus, the duration of observation in the present study was 12 weeks.

### Retinal and choroidal flow density

The macular perfusion changes after RRD surgery have not reached a commitment according to previous researches, which is due to different study designs and observation length. Wu et al. found significantly lower blood flow in both SCP and DCP than fellow eyes after retinal repair [26]. The surgical operation included vitrectomy and scleral buckling. The follow-up time was relatively longer than our research (3.6 ± 2.4 months, ranging 2–9 months). Wang et al., found significant increase in retinal blood flow in RRD eyes with successful PPV [22], however, the operation was combined with cataract surgery in their study. Phacoemulsification operation has been demonstrated to increase macular perfusion after surgery for up to 3 months [28, 29]. Furthermore, these two studies enrolled RRD patients with macular involvement. Detachment of macula before retinal repair could possibly influence macular perfusion even after successful reattachment [25]. Thus, the finding of differences in the alterations of various retinal blood flow among our macular-on RRD patients is more likely to present the exact fundus vasculature changes after surgery. For retinal blood flow, from 2 weeks to 6 weeks, we found an increasing trend in gas tamponade eyes (Tables 2 and 4). The short-term increase of blood flow might be related to postoperative inflammation [22]. On the other hand, we found a decrease of blood flow in SO tamponade eyes. More importantly, such opposite changing trend led to significant intergroup difference (Tables 2 and 4). From 6 weeks to 12 weeks, we could still found that SO tamponade led to more blood flow decrease in both SCPFD and DCPFD than Gas tamponade (Table 2). Thus, the present study demonstrated that SO tamponade may result in poorer macular perfusion in both superficial and deep retinal capillary plexus.

For postoperative choroidal status after retinal repair, previous studies used choroidal thickness as an indicator. Akkoyun et al. reported increased subfoveal choroidal thickness (SFCT) at 1 week after retinal repair, which they thought might represent inflammatory reaction [30]. Karimi et al. reported time-related reduce of SFCT in SO tamponade eyes which may possibly be due to tamponade effect of silicone oil [18]. However, change of choroidal thickness is not necessarily associated with choroidal vasculature change [31, 32]. We failed to find any significant result from choroidal thickness comparison during observation. On the other hand, we found significant intergroup difference in parafoveal CCPFD between Gas tamponade and SO tamponade eyes (Table 2). From 2 weeks to 6 weeks, parafoveal CCPFD had more increase in SO tamponade eyes. We suspect the increased of choroidal blood flow in SO group may indicate more severe postoperative choroidal inflammation, which could be related to the relatively greater amount of cryopexy or laser photocoagulation during procedure following SO use. We postulate that choroidal blood flow change could be a more sensitive indicator than choroidal thickness change.

### Retinal structural change

In previous studies on retinal structure change after retinal repair, most researchers divided the retinal layer in a relatively general way. In the present study, the retinal layers were strictly segmented into seven layers as introduced in the method section. As components of inner retina, both NFL and GCL + IPL have shown significant intergroup differences in their thicknesses changes. From 2 weeks to 6 weeks, SO tamponade led to more pronounced thickness decrease of these layers than gas tamponade (Tables 3 and 5). Such finding is consistent to the hypothesis of the toxic effect of SO on ganglion cell [16]. Kamila et al. also reported more pronounced decrease of thickness in GCL-IPL complex after SO tamponade in PPV, compared to gas (C3F8&SF6) tamponade [16]. As macular was not involved in our RRD patients, our findings may strongly indicate negative tamponade effects of silicone oil on inner retinal structures (NFL&GCL + IPL), which could possibly lead to poorer final visual acuity [23].

We found relatively more decrease of INL and OPL thicknesses in SO tamponade eyes than in Gas tamponade eyes during our observation (Table 3). Such findings seemed to contradict with results from certain previous studies. Yasin et al. reported thicker central INL and OPL in SO tamponade eyes than in fellow eyes [17]. Marcel et al. also reported a time related increase of INL-OPL thickness after successful retinal reattachment [23]. However, their patients all suffered from macular-off detachment. Theoretically, the inflammatory reactions after retinal detachment and reattachment repair may be observed in the pattern of increase in the volume of INL and OPL thickness [33]. However, in the present study, we found more decrease of these two retinal layers thicknesses in SO tamponade eyes than in Gas tamponade eyes, which led to significant intergroup difference (Table 3). We thought that in the process of structural remodeling of Müller cells after retinal detachment and reattachment, the INL and OPL layer thickness might also be influenced by factors other than postoperative inflammation. SO may have potential negative effects on INL and OPL in the undetached retinal area. Whether our finding indeed revealed variable impacts of different intravitreal tamponades deserve further studies.

Photoreceptor lies in the ONL + IS and OS+RPE layers. The integrity and thickness of IS/OS has been reported to be an important predictor of postoperative VA after RD surgery [34, 35]. On the other hand, due to ischemia in external layer and neuron cell loss, reduction of ONL thickness after retinal repair has been demonstrated, and maintenance of ONL thickness is also found to be correlated with postoperative VA [23, 24, 36–38]. We found that SO tamponade lead to more decrease in both ONL + IS and OS+RPE thicknesses (Table 3). We hypothesized that compared to gas tamponade, SO tamponade may lead to more thickness loss in ONL, and it might suppress photoreceptor recovery, which could both be related to poorer VA prognosis. Such finding might indicate potential negative impact of SO tamponade on visual prognosis.

During recent years, numerous researches has been performed for the underneath mechanism of Silicone Oil-Related Visual Loss (SORVL), and several pathophysiologic hypotheses have been proposed [39]. The following factors may all play their roles: photo-toxicity [40], fat soluble elements from the retina dissolved by SO [41], lost buffering capacity of the vitreous cavity and presence of intravitreal SO leading to impaired retinal homeostasis [5, 42, 43]. However, the exact underlying mechanism remains unknown.

Several remarks should be made with respect to the results of this study. On account of tamponade choice and timely SO removal clinical practice in our ophthalmic center, the observation time was relatively short and only comparison between gas and SO tamponade were analyzed. Extended observation period for SO tamponade impact would be necessary in future studies. Due to practical routine of intravitreal tamponade choice in our center, we only compared sterilized air and SO. We obviously lacked fundus vasculature and structure data before surgery as baseline, and we did not take fellow eyes data as control like several previous studies [16, 17, 22, 24, 26]. Unaffected eyes with asymptomatic feature could have vascular abnormalities in fundus diseases like retinal vein occlusion and primary open-angle glaucoma [44, 45]. Retinal detachment could also be bilateral in quite a number of patients. Thus, we did not take the fellow eye data of our patients as baseline or control to avoid potential bias. After all, the present study emphasized on the changes of fundus vasculature and structure during follow-up visits. Furthermore, the sample size of our study was relatively small.

## Conclusion

In conclusion, we investigated different intravitreal tamponade effects of silicone oil and gas on fundus vasculature and structure in macular-on rhegmatogenous retinal detachment patients underwent single pars plana vitrectomy. Silicone oil tamponade could lead to more macular abnormalities, which may be related to the ‘Silicone oil-related visual loss’.

## Data Availability

Available on reasonable request.

## Abbreviations

RRD: Rhegmatogenous retinal detachment
PPV: Pars plana vitrectomy
SO: Silicone oil
ILM: Inner Limiting Membrane
NFL: Nerve Fiber Layer
GCL: Ganglion Cell Layer
IPL: Inner Plexiform Layer
INL: Inner Nuclei Layer
OPL: Outer Plexiform Layer
ONL: Outer Nuclear Layer
IS/OS: Inner/Outer Segment
RPE: Retinal Pigment Epithelium
BRM: Bruch’s Membrane
SCPFD: Superficial capillary plexus flow density
DCPFD: Deep capillary plexus flow density
CCPFD: Choroidal capillary plexus flow density
OCT: Optical Coherence Tomography
OCTA: Optical Coherence Tomography Angiography
mm: Millimeter
